# Continuous renal replacement therapy for septic shock: practicalities for a pediatric intensivist

**DOI:** 10.1007/s00431-026-07290-x

**Published:** 2026-07-29

**Authors:** Lara Mary Titherington, Tommaso Bottesi, Francesco Guzzi, Stefano Romagnoli, Zaccaria Ricci

**Affiliations:** 1 Department of Health Sciences, Section of Anesthesiology and Intensive Care, University of Florence, Florence, Italy; 2 Department of Medical Specialties, Nephrology and Dialysis Unit, Nuovo Ospedale Santo Stefano, Azienda USL Toscana Centro, Prato, Italy; 3 Department of Anesthesia and Intensive Care, Azienda Ospedaliero Universitaria Careggi, Florence, Italy; 4 Department of Anesthesia and Critical Care, Meyer Children’s Hospital, IRCCS, Florence, Italy

**Keywords:** Hemofiltration, Pediatric septic shock, Continuous renal replacement therapy, Citrate anticoagulation

## Abstract

High-volume hemofiltration (HVHF) has been proposed as an adjunctive extracorporeal strategy for children with sepsis or septic shock requiring continuous renal replacement therapy (CRRT), based on the biological premise that enhanced convective clearance may attenuate circulating inflammatory mediator peaks. The 2026 Surviving Sepsis Campaign pediatric guidelines issued a conditional recommendation favoring HVHF over standard-volume hemofiltration in this setting, despite low-certainty evidence. This narrative review critically examines the biological rationale, available pediatric and adult evidence, and practical limitations of HVHF in pediatric septic shock. Although cytokines targeted by HVHF fall within the molecular-weight range theoretically amenable to clearance by high-flux membranes, clinical translation remains uncertain. Pediatric studies supporting HVHF are limited to small, single-center trials with heterogeneous dose definitions, variable treatment protocols, and fragile statistical estimates. In contrast, the largest observational pediatric datasets show no consistent dose–outcome relationship, while adult randomized trials and systematic reviews have failed to demonstrate survival benefit from higher-intensity or high-volume strategies. In small children, interpretation is further complicated by routine use of regional citrate anticoagulation, which may increase delivered effluent volume and blur the distinction between standard and high-volume prescriptions. Moreover, escalating CRRT dose has been potentially associated with dialytrauma, hemodynamic instability, micronutrient and amino acid losses, and clinically relevant antimicrobial underexposure.

*Conclusion*: Current evidence supports HVHF as biologically plausible but clinically unproven, with risks that should not be overlooked.

**Table:** 

**What is Known:** • *High-volume hemofiltration has a plausible biological rationale in pediatric sepsis, based on enhanced convective clearance of circulating inflammatory mediators*. • *The 2026 pediatric Surviving Sepsis Campaign guidelines recommended HVHF for children with sepsis or septic shock requiring renal replacement therapy*. • *The recommendation is labeled as “conditional” due to limited supporting evidence*.	
**What is New:** • *This review highlights that the pediatric evidence supporting HVHF is based on small single-center trials with heterogeneous definitions and statistically fragile estimates, while larger pediatric observational data and adult trials do not show a consistent dose-related clinical benefit*. • *Pediatric-specific factors, including regional citrate anticoagulation, high relative effluent doses in small children, and potential dialytrauma, may blur the distinction between standard and high-volume prescriptions and should temper routine clinical adoption*.

**What is Known:**

• *High-volume hemofiltration has a plausible biological rationale in pediatric sepsis, based on enhanced convective clearance of circulating inflammatory mediators*.

• *The 2026 pediatric Surviving Sepsis Campaign guidelines recommended HVHF for children with sepsis or septic shock requiring renal replacement therapy*.

• *The recommendation is labeled as “conditional” due to limited supporting evidence*.

**What is New:**

• *This review highlights that the pediatric evidence supporting HVHF is based on small single-center trials with heterogeneous definitions and statistically fragile estimates, while larger pediatric observational data and adult trials do not show a consistent dose-related clinical benefit*.

• *Pediatric-specific factors, including regional citrate anticoagulation, high relative effluent doses in small children, and potential dialytrauma, may blur the distinction between standard and high-volume prescriptions and should temper routine clinical adoption*.

## Introduction

Pediatric sepsis remains the leading cause of death in pediatric intensive care units worldwide. Despite sustained progress in early recognition, source control, antimicrobial stewardship, and protocolized resuscitation, mortality persists at roughly 25% in high-income countries and rises up to 40% in resource-limited settings [1]. Sepsis-associated acute kidney injury (SA-AKI) compounds this burden: contemporary epidemiology from the Assessment of Worldwide Acute kidney injury, Renal angina and Epidemiology (AWARE) cohort identifies AKI as an independent predictor of death in critically ill children, underscoring the prognostic weight of renal involvement [2].

Against this background, continuous renal replacement therapy (CRRT) has become the dominant modality of organ support in the critically ill child, increasingly deployed not only for fluid overload and metabolic derangement but also for the putative removal of circulating inflammatory mediators. Multinational registries describe a growing and heterogeneous population of children receiving CRRT [3, 4]. Survey data from European intensive care units reveal wide variability in prescribed effluent dose, anticoagulation, and timing [5, 6]. Furthermore, surveyed European centers showed to apply plasma exchange added to CRRT as the preferred blood-purification technique for septic shock and cytokine-storm syndromes, whereas high dose CRRT, also defined high volume hemofiltration (HVHF), is prevalently reserved for liver failure, and standard-dose CRRT is selected for rhabdomyolysis and intoxications [5].

Into this setting, the 2026 Surviving Sepsis Campaign pediatric guidelines introduced a conditional recommendation in favor of HVHF (> 35 mL/kg/h) over standard-volume hemofiltration (≤ 35 mL/kg/h) for all children with sepsis or septic shock requiring renal replacement therapy. Furthermore, the panel concluded that there is insufficient evidence to issue a recommendation on plasma exchange and extracorporeal blood purification in pediatric sepsis [7].

The present narrative review critically appraises the biological rationale for mediator clearance, scrutinizes the clinical evidence underpinning the dose threshold, and considers the contextual appropriateness of pediatric HVHF. It also aims to provide practical bedside considerations, identify key knowledge gaps, and outline a coherent research agenda.

## History, biological rationale, and theoretical advantages

The concept of HVHF emerged from experimental animal data demonstrating that ultrafiltration rates up to 6000 ml/h significantly improved hemodynamics in porcine endotoxemia models [8]. Translating this to clinical practice, a seminal trial by Ronco et al. demonstrated a survival benefit when increasing hemofiltration rates from 20 to 35 mL/kg/h, anchoring for many years this dosage as the standard of care of continuous renal replacement therapy (CRRT) in critically ill adult patients. This paper also suggested that septic patients could have received benefit by further increasing hemofiltration to 45 ml/kg/h and paved the way to the clinical application of HVHF [9].

To explain these findings, the “Peak Concentration Hypothesis” was introduced [10]. This model framework recognizes that sepsis is not a single-mediator, linear phenomenon, but rather a dynamic, non-selective dysregulation characterized by the simultaneous production of both pro-inflammatory (e.g., TNF-alpha, IL-1beta, IL-6, IL-8) and anti-inflammatory (e.g., IL-10, IL-1ra) cytokines (parallel theory). This represented a paradigm shift from the traditional sequential theory of cytokine dynamics, which posited that an initial systemic inflammatory response syndrome (SIRS) is followed chronologically by a compensatory anti-inflammatory response and subsequent immuno-paralysis [11]. Conversely, the parallel theory recognized that both inflammatory arms coexist from onset. Consequently, continuous, non-selective extracorporeal removal aims to blunt the peak concentrations of both components, thereby restoring immuno-homeostasis without entirely abolishing the necessary host immune response.

These target cytokines are middle-molecular-weight molecules (5 to 60 kDa), making them amenable to convective clearance, and membrane adsorption. HVHF would specifically maximize convective mass transfer via elevated ultrafiltration rates paired with high-volume replacement fluid, effectively leveraging the molecular-weight cutoffs of standard high-flux membranes which typically range from 30 to 40 kDa.

When applied to pediatric populations, this biological rationale presents distinct theoretical advantages. As pediatric patients possess a lower absolute circulating blood volume, they face a proportionally higher cytokine burden per kilogram of body weight, which frequently correlates with severe, rapid-onset hemodynamic instability [12]. This unique physiology suggests that children may derive an amplified clinical benefit from high-dose convective therapies compared to adults. Yet, no definitive trial has been published on this matter, to support high-level recommendations for clinical practice.

## Drawbacks, pitfalls, and available evidence

A primary obstacle in interpreting both the adult and pediatric literature is the absence of a standardized definition for HVHF. Thresholds vary across studies, with definitions encompassing effluent rates of > 35, > 45, > 50, or > 70 mL/kg/h [9, 13, 14]; this semantic heterogeneity precludes meaningful meta-analysis and leaves any specific threshold largely arbitrary. This concern is not merely semantic. Even within the evidence pooled for SSC Statement on blood purification, the standard-volume comparator arm of one constituent trial (35–50 mL/kg/h) lies largely above the > 35 mL/kg/h threshold by which the same statement defines HVHF [15].

In small children, this confounding concept is further amplified by the use of regional citrate anticoagulation. When standard diluted citrate solutions (18 mmol/L) are used, an additional prefilter infusion of 20 to 30 mL/kg/h is passively delivered as citrate solution, a background flow that frequently shifts an intended “standard” prescription directly into the HVHF range; if further convective or diffusive dose is not prescribed on top of this baseline citrate solution delivery, the child may be exposed to an excessive and potentially toxic systemic citrate load [16]. Because an aggregate effluent of 35 mL/kg/h is therefore routinely exceeded in ordinary pediatric practice, the rigid dichotomy of “35 versus > 35 mL/kg/h” advanced by the 2026 SSC may fail to separate two genuinely distinct treatment populations in the real world [7].

A further issue related to the lack of a clear pediatric HVHF definition is whether HVHF should necessarily be restricted to purely convective therapies, or whether a broader concept of high-volume CRRT may be more appropriate. Such a definition could require a minimum convective component, while still allowing part of the total effluent dose to be delivered by diffusion. This approach would better reflect contemporary pediatric CRRT practice, in which convective, diffusive, and mixed modalities are often used according to patient size, circuit performance, anticoagulation strategy, and local expertise.

Furthermore, real-world registry data sharpen concern related to the dose threshold. In the full Worldwide Exploration of Renal Replacement Outcomes Collaborative in Kidney Disease (WE-ROCK) cohort of 980 children and young adults, the prescribed median of 41.9 (IQR 30.9–59.9) mL/kg/h in this population [3] sit well above the 20–25 mL/kg/h standard indicated by KDIGO [17] and underscored that routine pediatric practice already operates within the HVHF range by default. Furthermore, the prescribed dose per kilogram did not differ between survivors and non-survivors to ICU discharge (41.2 vs. 45.5 mL/kg/h; p = 0.07), and neither dose category nor effluent volume indexed to body surface area were different in the two groups (*p* = 0.2 and *p* = 0.3) and, tendentially, the recorded dose was numerically higher among decedents [3].

The sepsis-specific secondary analysis of the WE-ROCK database reproduced this dissociation. Of 1,016 patients, 446 (44%) were septic at initiation and fared substantially worse outcomes than their non-septic counterparts: in-hospital mortality 47% vs. 31%; major acute kidney events at 90 days (MAKE-90) 70% vs. 61%. Among the 393 septic patients surviving beyond 48 h, the prescribed dose over the first week of CRRT was indistinguishable between those who did and did not reach MAKE-90 (43 vs. 45 mL/kg/h; *p* = 0.92), as was the dose over the first two days (*p* = 0.74). The authors proposed that worse outcomes were not explained by insufficient dialysis dose, but were instead associated with the cumulative duration of vasoactive support during CRRT, a variable proposed by the authors as a possible consequence of dialytrauma. This duration was independently associated with both MAKE-90 (adjusted OR 1.16 per additional day; 95% CI 1.05–1.28) and mortality (adjusted OR 1.20 per additional day; 95% CI 1.10–1.32) [4].

The dose paradox is particularly evident in the smallest patients. Patients below 10 kg of weight, according to WE-ROCK data, received a median dose of 63.8 mL/kg/h (IQR 49.2–88.1) [18]. When baseline doses in children weighing less than 10 kg were stratified by tertile (< 53, 53–80, and > 80 mL/kg/h), the youngest, smallest infants routinely received the highest relative doses, yet those exposed to an initial dose > 80 mL/kg/h experienced the highest rates of RRT dependence at hospital discharge and the longest durations of mechanical ventilation [18].

These comparisons concern the prescribed rather than the delivered dose, which WE-ROCK did not capture, a caveat that tempers but does not overturn the inference. Across the largest contemporary multinational pediatric CRRT cohort, outcomes were associated with illness severity and a marker of iatrogenic hemodynamic instability more than with the magnitude of the prescribed dose.

Beyond the absence of efficacy data, escalating the CRRT dose carries distinct clinical costs. High ultrafiltration rates accelerate the non-selective clearance of essential micronutrients, water-soluble vitamins, and amino acids [19]. Of greater concern, high-turnover convective regimens accelerate the removal of hydrophilic antimicrobials with low protein binding and a small volume of distribution, including β-lactams, aminoglycosides, and vancomycin, because their extracorporeal clearance scales largely with the ultrafiltration rate. Although the magnitude varies across agents, the net result is a clinically relevant risk of antibiotic underdosing in precisely the window in which optimized exposure is most decisive for outcome [20]. Notably, the limited pediatric studies that suggested a survival benefit did not systematically monitor or report these metabolic and pharmacological trade-offs. β-lactams and other hydrophilic agents are significantly vulnerable to enhanced extracorporeal clearance, potentially resulting in subtherapeutic exposure unless dosing is individualized and therapeutic drug monitoring is available [20].

These observational data do not establish that higher doses worsen outcomes, but neither do they support a dose-responsive survival benefit; rather, they raise the possibility that the (high) doses already delivered to these infants incur the costs of dialytrauma and micronutrient loss without a commensurate survival gain.

## The 2026 pediatric SSC

The pediatric recommendation on HVHF was modified between the 2020 and 2026 iterations of the SSC. In 2020 the panel suggested against HVHF, citing the absence of demonstrated benefit and a possible signal for hyperglycemia in a single observational pediatric study [21, 22]. In 2026, the pediatric Surviving Sepsis Campaign issued a conditional, low-certainty recommendation in favor of HVHF (> 35 mL/kg/h) for children with sepsis or septic shock requiring renal replacement therapy. This position likely followed several pediatric-specific considerations. Contemporary CRRT platforms, including newer-generation devices and pediatric-dedicated circuits, have made extracorporeal therapies technically more feasible in smaller children, potentially allowing safer delivery of higher effluent doses and enhanced solute clearance. In addition, current pediatric CRRT practice frequently involves prescribed doses above 35 mL/kg/h, closing the gap, in routine clinical practice, between “standard” and “high-volume” prescriptions. Finally, the biological rationale for mediator clearance may appear especially compelling in children, whose lower absolute circulating blood volume could translate into a proportionally greater inflammatory mediator burden per kilogram of body weight. Nevertheless, guideline recommendations must ultimately rest on the strength and consistency of the available clinical evidence, which remains limited in this field. The 2026 recommendation was based on a de novo, panel-conducted meta-analysis of three single-center randomized controlled trials enrolling a combined 195 children with sepsis, which reported lower mortality with HVHF than with standard-volume hemofiltration (RR 0.58; 95% CI 0.34–0.98; p = 0.04) [7, 15, 23, 24]. Although this pooled estimate supports the biological plausibility of HVHF and justifies further investigation, it should be interpreted with caution because of important limitations in the underlying evidence. All three trials were conducted at single nation centers, delivered continuous venovenous hemofiltration without a diffusive component, and were characterized by small samples and low absolute event counts; they were further heterogeneous in their operational definitions of HVHF (50–70, 50–100, and 60 mL/kg/h for Cui 2015, Meng 2018 and Ning 2020, respectively) and in the timing and duration of therapy [15, 23, 24]. The panel itself acknowledged that this configuration left the pooled estimate susceptible to type I error. Because two of the trials [15, 23] antedate the 2020 guideline and the third [24] is contemporaneous with it, the revised recommendation reflects a reappraisal of largely pre-existing data rather than the incorporation of new, higher-quality evidence. An independent pediatric review by Bhatt et al. [12], pooling an overlapping but non-identical trial set [15, 21, 24] (total cohort *n* = 278), reached a similar but non-identical estimate (RR 0.60; 95% CI 0.39–0.93): in this work, the largest pediatric dataset, the retrospective cohort of Miao et al. (*n* = 155), was neutral and found a higher incidence of hyperglycemia in the HVHF group [21]. Crucially, none of these findings has been tested in a multicenter trial (Table 1). These limitations do not negate the clinical rationale behind the recommendation, but they suggest that the current evidence is better suited to support research prioritization than routine adoption across heterogeneous pediatric CRRT populations.

**Table 1:** Studies comparing high volume to low volume hemofiltration in pediatric sepsis

Study	Design	*N*	HVHF/SVHF definitions	Studies modality	Primary endpoint	Mortality outcome (28-day)	Key biases and critical limitations
** Cui 2015**[23]	Single-center, prospective randomized trial	72 (34 HVHF, 38 SVHF)	**HVHF:** 50–70 mL/kg/h < br > **SVHF:** 35 mL/kg/h	CVVH	28-day mortality	**29.4% vs. 36.8%** (*P* = 0.50); no statistical difference	Small sample size; single-center; evolution of sepsis care over the 5-year study period
** Ning 2020**[24]	Prospective, randomized, open-label trial	47 (25 HVHF, 22 Control)	**HVHF:** 60 mL/kg/h < br > **Control:** 30 mL/kg/h	CVVH	28-day mortality	**20.0% vs. 40.9%** (*P* = 0.201); no statistical difference	Very small sample size (preliminary data); open-label design (lack of blinding)
** Meng 2018**[15]	Randomized controlled trial	76 (38 HVHF, 38 Control)	**HVHF:** 50–100 mL/kg/h < br > **Control:** 35–50 mL/kg/h	CVVH	Safety and effectiveness (Composite)	**7.9% vs. 26.3%** (*P* = 0.033); **statistically significant reduction**	Strict exclusion criteria (patients with heart, lung, liver, or brain dysfunction excluded); insufficient specific observation indices
** Miao 2018**[21]	Retrospective cohort study	155 (93 HVHF, 62 CVVH)	**HVHF:** 50–70 mL/kg/h (mean 63.5) < br > **CVVH:**35 mL/kg/h	CVVH	Normalization of temp and improved liver function	**24.7% vs. 33.8%** (*P* = 0.216); no statistical difference	Retrospective nature (selection bias); baseline severity imbalance (HVHF group had significantly more pneumonia and ARDS)

A formal fragility analysis provides an additional perspective regarding robustness. Applying the meta-analytic Fragility Index [25], an extension of the original single-trial Fragility Index concept [26], to the random-effects estimate of the panel-conducted meta-analysis, we found that the conversion of a single survivor to a non-survivor in the HVHF arm of the most heavily weighted trial (itself individually non-significant) [23] would shift the pooled 95% CI to include unity (Fragility Index = 1; Fragility Quotient = 0.005); the result remained fragile under a fixed-effect model (supplementary material).

The adult literature offers a further useful comparator. Although an early positive signal for higher-dose continuous hemofiltration emerged in critically ill adults with acute kidney injury [9], adequately powered adult trials have since failed to demonstrate a survival benefit of higher-intensity or high-volume strategies in AKI or septic shock [14, 27]. Systematic reviews have reached concordant, neutral conclusions (pooled 28-day mortality RR ≈ 0.96) [28], and no current adult guideline recommends HVHF for sepsis [29]. Some biological justification exists for expecting that a convective strategy unsuccessful in adults may behave differently in children. This rationale rests on pediatric-specific factors, including a proportionally smaller circulating blood volume relative to the extracorporeal circuit, distinct cytokine clearance kinetics, and developmental differences in immune response. These arguments are plausible but remain hypothesis-generating and unconfirmed in pediatric trials; narrative syntheses of the pediatric literature likewise characterize HVHF as promising while emphasizing the need for larger confirmatory studies [12].

**Fig. 1:**
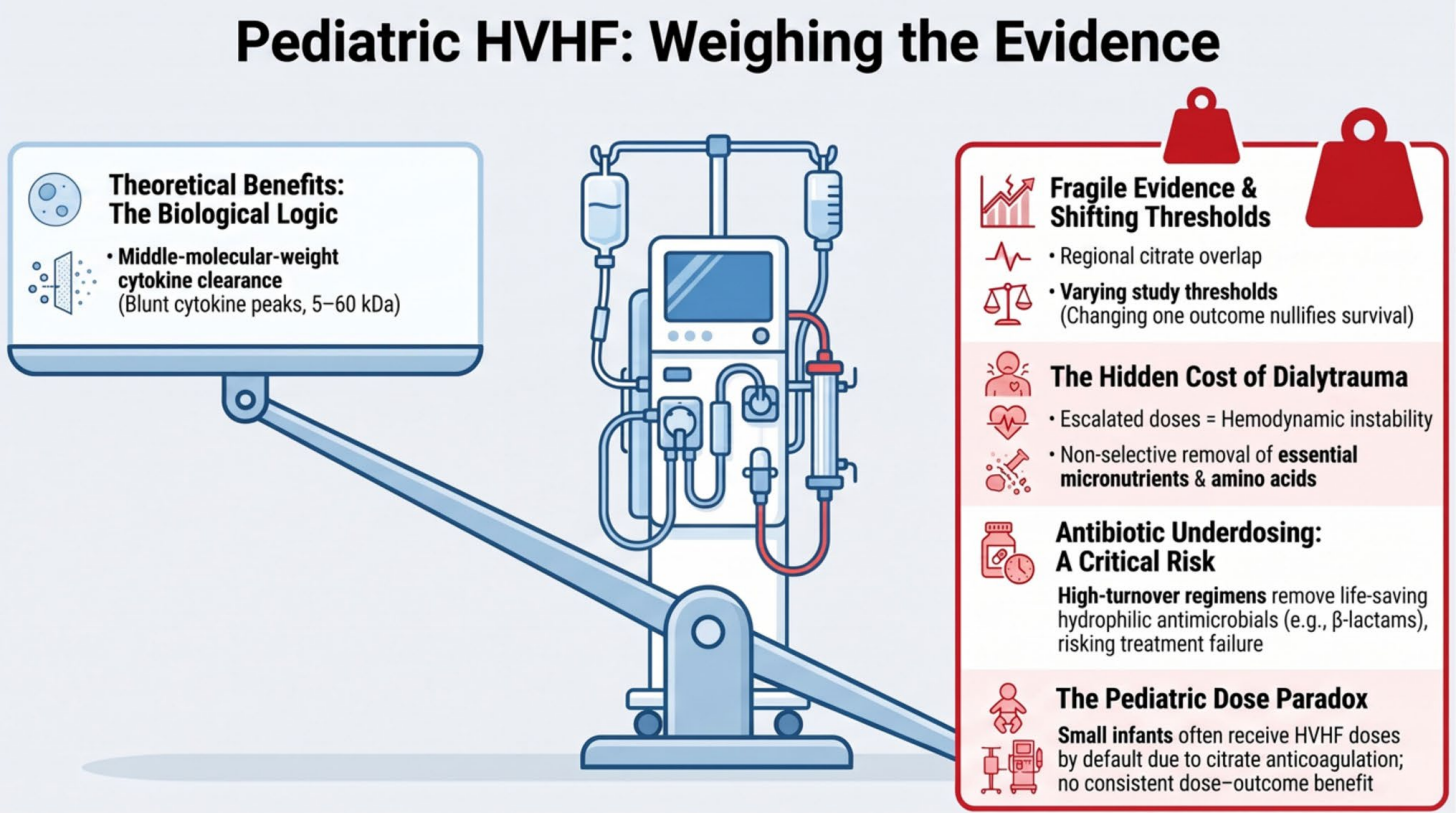
Conceptual balance of high-volume hemofiltration in pediatric septic shock

Taken together, the current evidence consists of small single-center trials with statistically fragile estimates, a large neutral pediatric retrospective cohort, real-world data showing that children already receive CRRT doses above “standard” thresholds, and consistently neutral adult data. Consequently, we can consider HVHF as a biologically plausible but clinically unproven intervention (Fig. 1). The 2026 SSC statement should therefore be read more as a call to prioritize research on pediatric HVHF than as a directive for routine clinical practice. Such a research agenda should begin with a pragmatic, pediatric-specific definition of HVHF, aligned with contemporary CRRT practice and able to distinguish intentional high-volume therapy from effluent volumes already generated by routine prescriptions, particularly in small children receiving regional citrate anticoagulation.

## What should clinicians do tomorrow morning?

In the absence of definitive evidence supporting routine HVHF in pediatric septic shock, clinicians should resist the temptation to equate “more hemofiltration” with better care. The timing of CRRT initiation should be early enough to address fluid overload, metabolic derangements, and hemodynamic consequences of acute kidney injury, but should not be driven solely by the intention to enhance inflammatory mediator clearance in the absence of clear clinical indications. Available literature on timing associates the CRRT start in the first 24–48 h after PICU admission with improved outcomes [30]. For most children requiring CRRT, the safest and most defensible approach remains the prescription of a standard CRRT dose, adjusted to the clinical goal and reassessed frequently according to delivered rather than merely prescribed clearance. Excessive dose escalation (i.e., above 35–40 ml/kg/h) should be avoided unless there is a specific, individualized indication, because higher effluent rates may increase hemodynamic stress, micronutrient and amino acid losses, and unintended antimicrobial clearance [19, 20]. In septic children, antibiotic optimization is likely to be more immediately relevant than empiric intensification of hemofiltration: therefore, dosing should be individualized according to residual kidney function, CRRT modality, effluent rate, protein binding, volume of distribution, and severity of illness. In many cases, this means avoiding automatic dose reduction solely because CRRT has been initiated, and instead maintaining the initial regimen recommended for patients with preserved renal function, at least during the early phase of sepsis and until drug exposure can be reassessed [31]. Whenever available, therapeutic drug monitoring should be used, especially for β-lactams, vancomycin, and aminoglycosides, with early adjustment to avoid subtherapeutic exposure during the critical first days of sepsis [31]. Regional citrate anticoagulation should also be prescribed carefully. In small children, low-dose, concentrated citrate solutions may help limit unintended prefilter volume delivery and avoid pushing an apparently standard prescription into the high-volume range simply through anticoagulation strategy [16]. Citrate balance, ionized calcium, total-to-ionized calcium ratio, acid–base status, and electrolyte trends should be monitored closely. Ultimately, CRRT prescription in pediatric septic shock should be individualized rather than protocolized: dose, modality, anticoagulation, and antimicrobial strategy should be tailored to patient size, hemodynamic tolerance, inflammatory phenotype, metabolic needs, and drug exposure. Tomorrow morning, the practical message is not to ignore HVHF, but to use CRRT deliberately, avoiding both therapeutic nihilism and dose escalation by reflex (Box 1).

**Box 1** Practical bedside implications

**Table:** 

• Timing of CRRT initiation should not be delayed in severe AKI if signs of fluid overload are present. Typically 24 or 48 h after PICU admission is the ideal timing • Start with a standard CRRT prescription (35–40 ml/kg/h) • A universally accepted pediatric definition of HVHF is currently lacking, with proposed thresholds varying across studies and practice settings, commonly falling within an approximate range of 40–80 mL/kg/h • Avoid excessive effluent-dose escalation unless a clear individualized indication exists • Optimize antimicrobial dosing according to CRRT modality, effluent rate, residual kidney function, and illness severity • In high volume CRRT prescriptions, convective clearance should represent the predominant component of the total effluent dose, accounting for approximately 50–80% of the overall prescription • Use therapeutic drug monitoring whenever available, particularly for β-lactams, vancomycin, and aminoglycosides • Prescribe regional citrate anticoagulation cautiously, preferably using low-dose, concentrated citrate strategies in small children • Monitor ionized calcium, total-to-ionized calcium ratio, acid–base balance, electrolytes, and calcium requirements: in case of HVHF monitoring frequency should be increased • Individualize CRRT dose, modality, anticoagulation, and drug dosing to patient size, hemodynamic tolerance, metabolic needs, and treatment goals **Practical message:** pediatric CRRT in septic shock should prioritize precision, safety, and antimicrobial adequacy over reflex dose escalation	

## Future perspectives

Resolving the uncertainty about pediatric HVHF demands a research agenda that moves beyond the aggregation of small, single-center experiences. The foremost priority is an adequately powered, multinational randomized trial that prospectively standardizes the HVHF (or high-volume CRRT) definition, explicitly separates the prescribed convective or diffusive dose, and stratifies enrollment by body weight to address the disproportionate exposure of the smallest infants. Such a trial should be designed around clinically meaningful, patient-centered endpoints, MAKE-90, kidney recovery, ventilator-free days, and long-term neurodevelopmental outcomes, rather than short-term mortality alone, given the low absolute event counts that render mortality-based estimates statistically fragile. Adaptive or Bayesian designs may offer efficiency in this rare, heterogeneous population. Anticoagulation strategies should be included in the trial methodology.

Embedded translational sub-studies would materially strengthen any future trial. Serial cytokine kinetics could test the peak-concentration hypothesis directly in children, while systematic pharmacokinetic sampling, therapeutic drug monitoring of hydrophilic antimicrobials, and quantification of amino acid and micronutrient losses would characterize the dialytrauma that current efficacy studies have largely overlooked.

Finally, the relationship between high-volume treatments and modern adsorptive technologies merits formal evaluation. As selective hemoadsorption cartridges increasingly provide targeted mediator removal, future studies should clarify whether nonselective high-volume ultrafiltration retains an independent role, or whether enrichment strategies, identifying phenotypes such as thrombocytopenia-associated multiple organ failure or hyperammonemia most likely to benefit, offer a more rational path than uniform dose escalation across an undifferentiated septic population.

## Conclusions

The 2026 Surviving Sepsis Campaign conditional endorsement in favor of HVHF rests on a narrow and fragile evidentiary base. This pediatric signal stands in contrast to a uniformly neutral adult literature, in which adequately powered trials and systematic reviews have repeatedly failed to demonstrate benefit, and sits uneasily beside the pediatric guideline’s lack of support to mechanistically related blood-purification strategies. Real-world registry data add a further paradox: the smallest infants already receive doses well within the high-volume range with no discernible dose–outcome gradient, suggesting that prevailing practice may incur the metabolic and pharmacological costs of dialytrauma without a commensurate survival gain.

The available evidence supports HVHF as a biologically plausible but clinically unproven strategy, with potential risks that should not be overlooked. The challenge for adequately powered, multicenter trials with standardized dosing and patient-centered endpoints will no longer be simply to deliver more hemofiltration, but to deliver the right hemofiltration to the right patient at the right time.

## Data Availability

No datasets were generated or analysed during the current study.
